# RIPK3 promotes adenovirus type 5 activity

**DOI:** 10.1038/s41419-017-0110-8

**Published:** 2017-12-13

**Authors:** Melanie Weigert, Alex Binks, Suzanne Dowson, Elaine Y. L. Leung, Dimitris Athineos, Xinzi Yu, Margaret Mullin, Josephine B. Walton, Clare Orange, Darren Ennis, Karen Blyth, Stephen W. G. Tait, Iain A. McNeish

**Affiliations:** 1 Institute of Cancer Sciences, University of Glasgow, Garscube Estate, Glasgow, G61 1QH UK; 2 Cancer Research UK Beatson Institute, Glasgow, UK; 3 School of Life Sciences, University of Glasgow, Glasgow, UK

## Abstract

Oncolytic adenoviral mutants infect human malignant cells and replicate selectively within them. This induces direct cytotoxicity that can also trigger profound innate and adaptive immune responses. However, the mechanism by which adenoviruses produce cell death remains uncertain. We previously suggested that type 5 adenoviruses, including the E1A CR2 deletion mutant *dl*922-947, might induce a novel form of programmed death resembling necroptosis. Here we have investigated the roles of core necrosis proteins RIPK1, RIPK3 and MLKL in the cytotoxicity of *dl*922-947 and other adenovirus serotypes. By electron microscopy, we show that *dl*922-947 induces similar necrotic morphology as TSZ treatment (TNF-α, Smac mimetic, zVAD.fmk). However, *dl*922-947-mediated death is independent of TNF-α signalling, does not require RIPK1 and does not rely upon the presence of MLKL. However, inhibition of caspases, specifically caspase-8, induces necroptosis that is RIPK3 dependent and significantly enhances *dl*922-947 cytotoxicity. Moreover, using CRISPR/Cas9 gene editing, we demonstrate that the increase in cytotoxicity seen upon caspase inhibition is also MLKL dependent. Even in the absence of caspase inhibition, RIPK3 expression promotes *dl*922-947 and wild-type adenovirus type 5 efficacy both in vitro and in vivo. Together, these results suggest that adenovirus induces a form of programmed necrosis that differs from classical TSZ necroptosis.

## Introduction

Oncolytic viruses are a promising new therapy for cancer. They can infect cancer cells, multiply selectively within them and cause cell death, with release of mature viral particles that infect neighbouring cells. We have previously shown that the E1A CR2-deleted adenovirus type 5 mutant *dl*922-947 has considerable activity in ovarian cancer and is more potent than both E1A wild-type adenoviruses and the E1B-55K mutant *dl*1520 (Onyx-015)^1,2^. We have also shown that *dl*922-947 induces robust DNA double-strand break damage in infected cells^3^ and utilises components of the homologous recombination pathway to promote efficacy^4^.

The exact mechanisms by which adenoviruses cause cell death remain uncertain. We previously showed that classical apoptosis is not the primary mode of cell death following E1A CR2-deleted adenovirus infection in ovarian cancer and that autophagy was likely to be a survival mechanism^5^. Our overall conclusion was that adenovirus cytotoxicity had the features of a programmed necrotic process.

Necrosis is now realised to be a highly regulated form of cell death^6^. The best-characterised pathway of programmed necrosis (or necroptosis) occurs when tumour necrosis factor (TNF)-α (T) binds to TNFR1 in the presence of a Smac-mimetic (S) and the pan-caspase inhibitor zVAD.fmk (Z). The critical step in TSZ-induced necroptosis is the formation of the necrosome, a complex that includes RIPK1 and RIPK3, both of which contain RHIM (RIP homotypic interaction motif) domains. Mixed lineage kinase domain-like (MLKL) then binds to RIPK3 through its C-terminal kinase-like domain, which is phosphorylated at T357/S358 by RIPK3, leading to its activation and translocation to the cell membrane, where it is involved in the formation of pores, ion influx and membrane disruption^7,8^. Other pathways of programmed necrotic death exist that are independent of the TNF-α/TNFR interaction—Tenev et al. and Feoktiskova et al. described the ripoptosome, a death-inducing complex that forms upon genotoxic stress and that leads to caspase-dependent apoptosis or caspase-independent necrosis depending upon both cellular caspase-8 activity^9,10^ and the levels and cleavage state of cFLIP (reviewed in ref.^11^). Similarly, necrosome-like complexes can also form as a result of Toll-like receptor (TLR) signalling^12^ in response to lipopolysaccharide^13^ and poly(I:C)^14^. In addition, RIPK3 can induce necrosis independently of RIPK1 and MLKL; for example, following ischaemia/reperfusion injury or doxorubicin treatment, RIPK3 induces necrotic death in cardiac myocytes by binding directly to and activating CaMKII (calcium-calmodulin-dependent protein kinase)^15^.

Multiple viruses trigger RIPK1/RIPK3-dependent responses, including vaccinia^16,17^, murine cytomegalovirus^18^ and influenza A^19^. In herpes simplex virus (HSV)-1 infection, ICP6 binds to RIPK1/RIPK3 in both human and murine cells, which triggers a necrotic response in mouse^20^ but suppresses necrosis in human cells^21^.

Here we have investigated the roles of RIPK1, RIPK3 and MLKL in the cytotoxicity of *dl*922-947 and other adenovirus serotypes. We show that adenovirus induces similar necrotic morphology to TSZ. However, cytotoxicity is independent of TNF-α signalling and RIPK1 and does not rely upon the presence of MLKL. RIPK3 expression augments adenovirus efficacy, while caspase-8 inhibition can significantly enhance activity in a RIPK3- and MLKL-dependent manner. These results suggest that adenovirus induces a form of programmed necrosis that differs from classical TSZ necroptosis.

## Results

### Induction of necroptosis in tumour cells

**Fig. 1:**
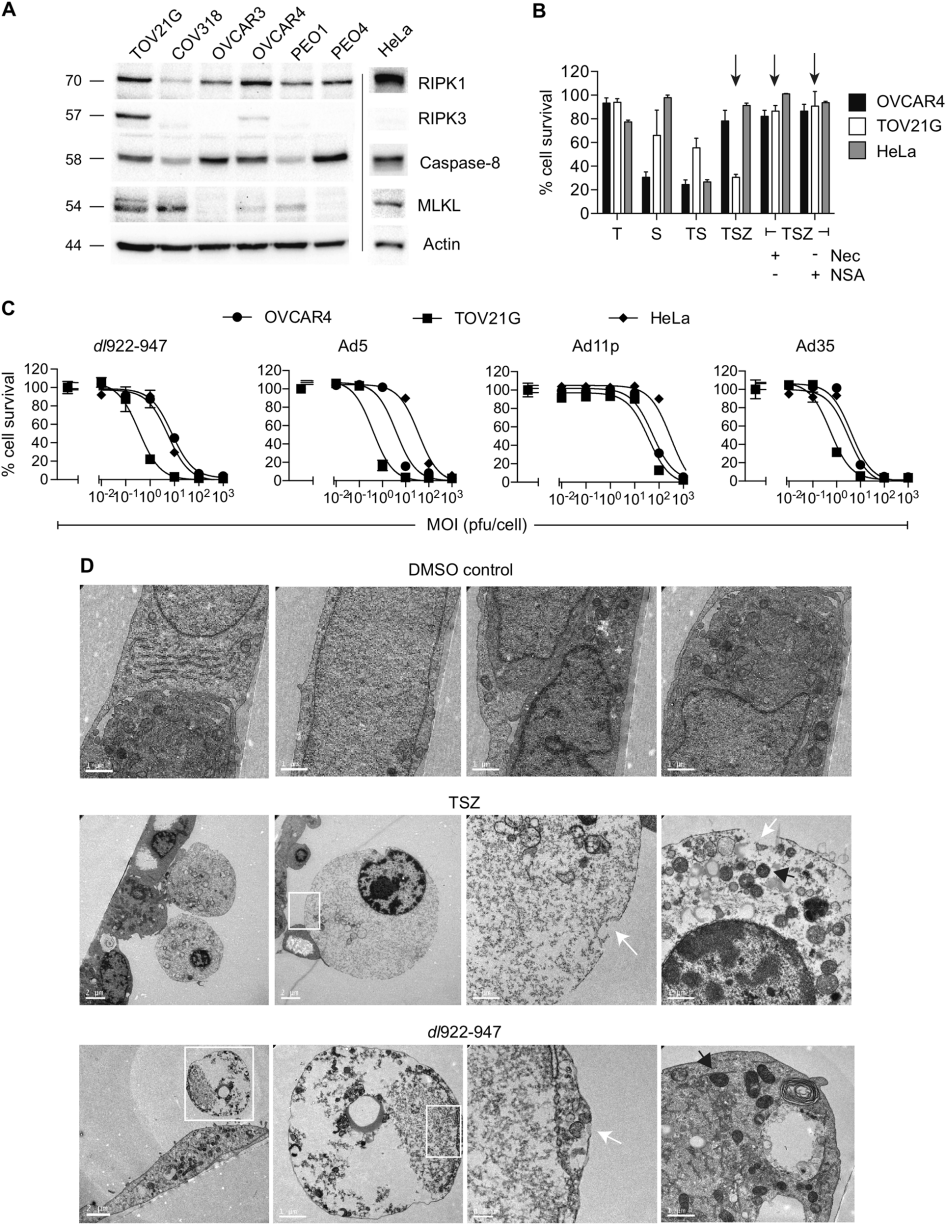
Induction of necrosis in ovarian cancer cells **a** Expression of RIPK1, RIPK3, caspase-8 and MLKL was assessed in ovarian cancer cells and HeLa by immunoblot. **b** OVCAR4, TOV21G and HeLa cells were treated with TNF-α (T, 20 ng/ml), the Smac-mimetic LCL-161 (S, 1 μM) and/or zVAD.fmk (Z, 25 µM) for 6 h in the presence and absence of necrostatin-1 (Nec, 10 µM) and necrosulfonamide (NSA, 1 µM). Arrows indicate necrosis induced by TSZ in TOV21G cells, reversed by Nec and NSA. Cell survival was assessed by MTT assay. **c** OVCAR4, TOV21G and HeLa cells were infected with *dl*922-947, Ad5, Ad35 and Ad11p (MOI 0.01–1000 pfu/cell) for 120 h. Cell survival was assessed by MTT assay. **d** Transmission electron microscopic images of TOV21G cells following 6 h treatment with TSZ (concentrations as 1B) or 48 h infection with *dl*922-947 (MOI 1). White arrows indicate sites of membrane rupture; black arrows indicate electron-dense mitochondria

We first investigated the expression of RIPK1, RIPK3, caspase-8 and MLKL in a panel of ovarian cancer cell lines as well as HeLa cells (Fig. 1a). Only TOV21G cells expressed all three proteins in equal quantities and were sensitive to necroptosis induced by TSZ (TNF-α, Smac mimetic, zVAD.fmk) that was reversible by treatment with necrostatin-1 and necrosulphonamide (NSA) (Fig. 1b). We evaluated the sensitivity of TOV21G and two TSZ-resistant lines, OVCAR4 and HeLa, to the E1A CR2-deleted Ad5 vector *dl*922-947 as well as wild-type adenovirus of different serotypes (Fig. 1c). As we have noted previously^22^, TOV21G was consistently the most sensitive line. By electron microscopy, the morphology of *dl*922-947-treated cells was similar to morphology of those treated with TSZ, with swollen mitochondria, cytoplasmic disintegration and loss of membrane integrity (Fig. 1d, Fig. S1), in keeping with a necrotic process. In addition, assessment by flow cytometry with a membrane-impermeable fluorescent dye showed progressive loss of membrane integrity following *dl*922-947 infection in both TOV21G and OVCAR4 cells (Fig. S2)

### *dl*922-947-induced necrosis does not result from local TNF-α production nor is RIPK1 involved

**Fig. 2:**
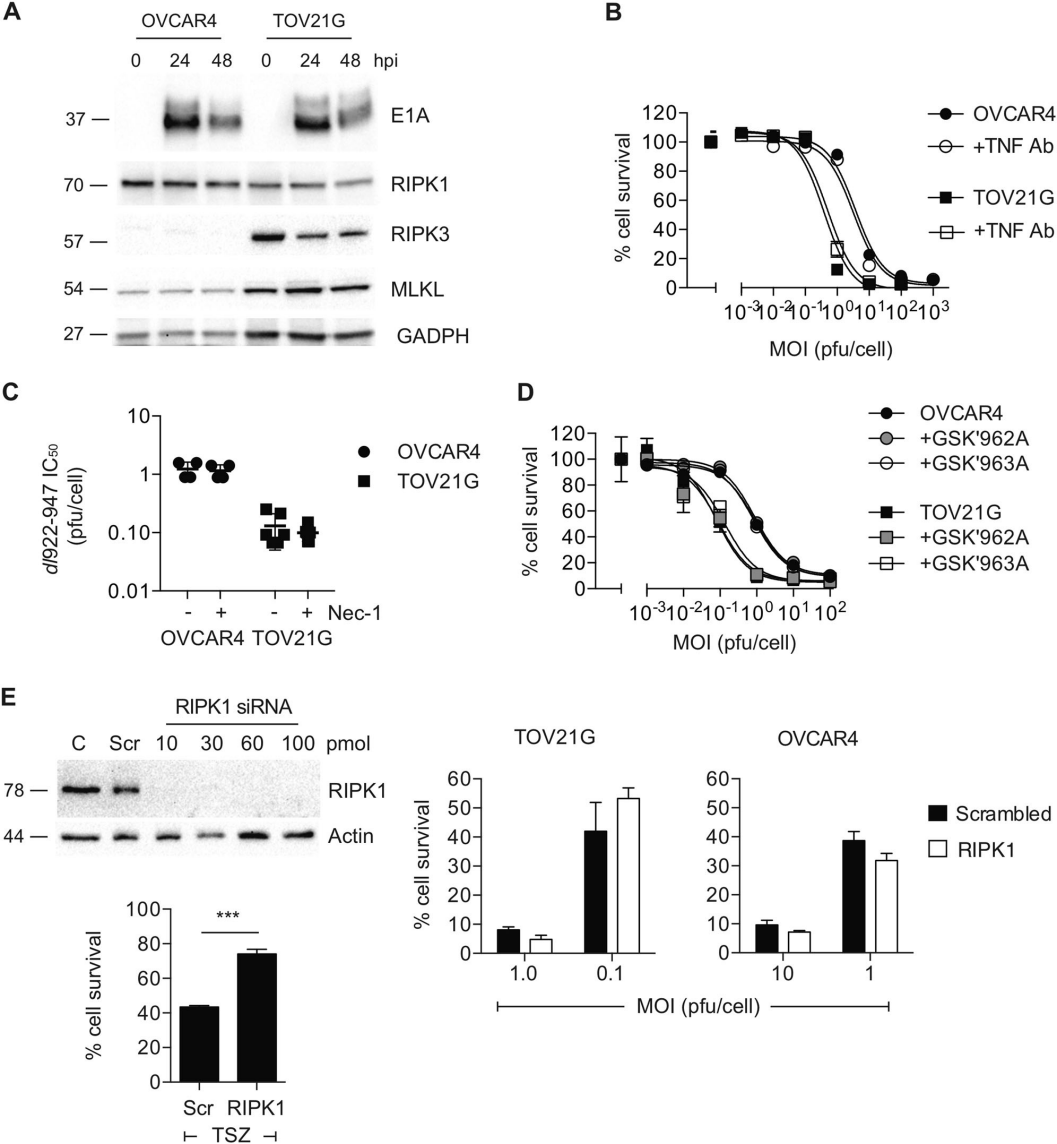
RIPK1 plays no role in *dl*922-947-induced cytotoxicity **a** Expression of core necrosis proteins RIPK1, RIPK3 and MLKL following *dl*922-947 infection was assessed in OVCAR4 (MOI 10) and TOV21G (MOI 1) cells. **b** OVCAR4 and TOV21G cells were infected with *dl*922-947 in the presence and absence of blocking anti-TNF-α Ab (1 µg/ml). Cell survival was assessed after 120 h by MTT assay. **c** Necrostatin-1 (10 µM) treatment has no effect on *dl*922-947 cytotoxicity in OVCAR4 and TOV21G cells. Each dot represents IC_50_ from a single triplicate experiment. **d** OVCAR4 and TOV21G cells were infected with *dl*922-947 in the presence and absence of blocking RIPK1 inhibitors GSK3002962A and GSK2003963A (both 10 nM). Cell survival was assessed after 120 h by MTT assay. **e** RIPK1 knockdown in TOV21G cells (left) 24 h following transfection with 10–100 pmol siRNA. 10 pmol RIPK1 siRNA significantly reduced TSZ-induced cell death. Adenovirus cytotoxicity experiments (right) utilised 10 pmol siRNA. Cells were infected with *dl*922-947 24 h following siRNA transfection. Cell survival was assessed 120 h thereafter

Infection with *dl*922-947 was not associated with marked changes in the expression of RIPK1, RIPK3, MLKL (Fig. 2a) or caspase-8 (Fig. S3). Inhibition of TNF-α using a blocking antibody had no effect on *dl*922-947 activity (Fig. 2b), while treatment with necrostatin-1 (Fig. 2c, Fig. S4) and the RIPK1 inhibitors GSK3002962A and GSK3002963A (Fig. 2d, Fig. S5) also had no effect on virus efficacy. Finally, small interfering RNA (siRNA)-mediated RIPK1 knockdown also did not inhibit *dl*922-947 activity (Fig. 2e). Thus we conclude that virus-induced death does not rely upon either TNF-α or RIPK1.

### MLKL is not an absolute requirement for *dl*922-947-induced cytotoxicity

**Fig. 3:**
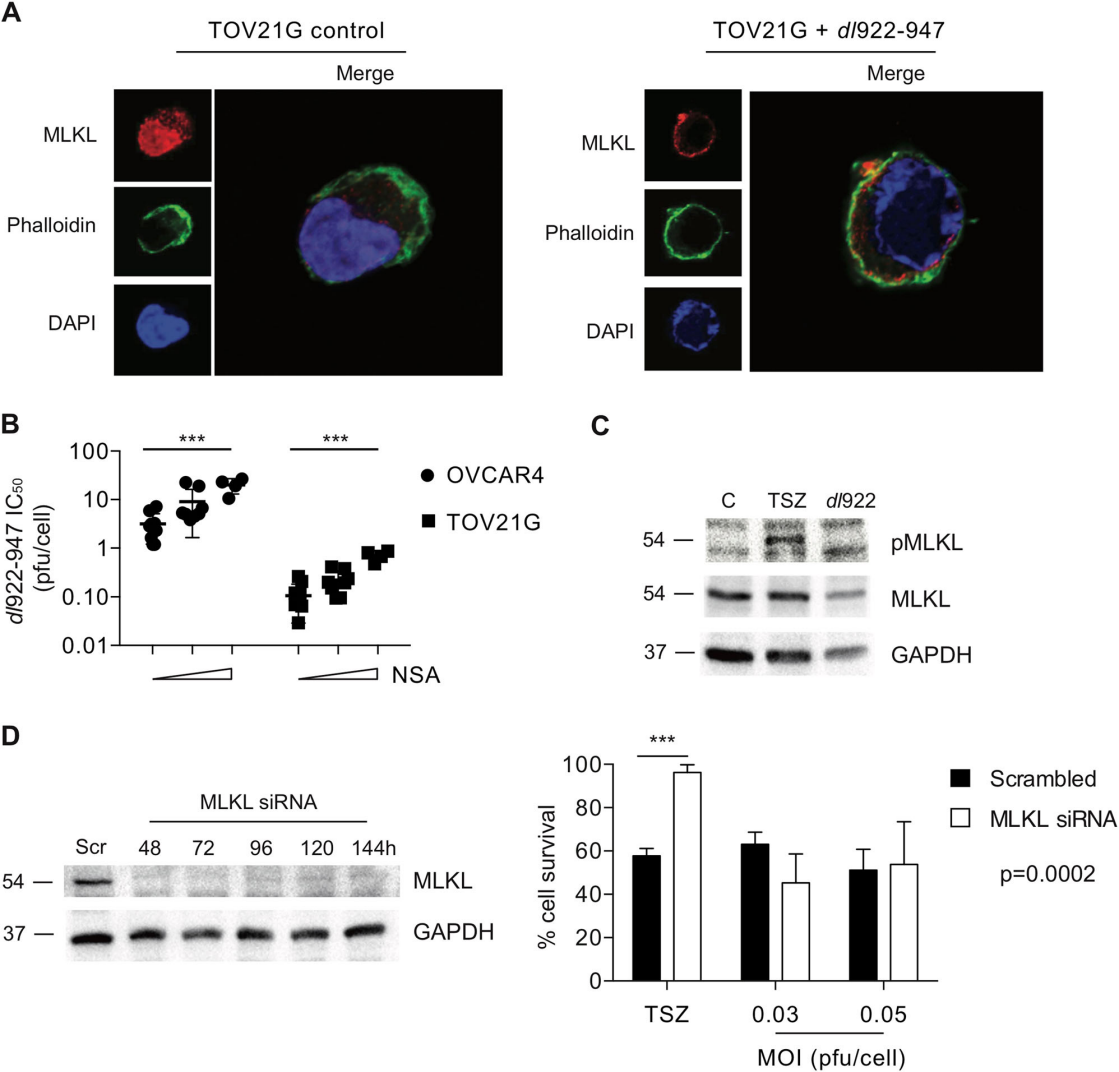
MLKL is not essential for *dl*922-947-induced cytotoxicity **a** MLKL relocalises to the plasma membrane in TOV21G following *dl*922-947 infection (MOI 1, 48 h). **b** Necrosulphonamide (NSA) treatment significantly inhibits *dl*922-947 cytotoxicity in OVCAR4 and TOV21G cells. Each dot represents IC_50_ from a single triplicate experiment. NSA concentrations were 0, 3 and 10 µM. ***p < 0.001 for linear trend across NSA concentrations. **c** MLKL phosphorylation was assessed in TOV21G cells following TSZ (6 h) or *dl*922-947 (MOI 1, 48 h) treatment by immunoblot. **d** MLKL knockdown in TOV21G cells (left) up to 144 h following transfection with 10 pmol siRNA. Adenovirus and TSZ cytotoxicity experiments (right). Cells were treated with TSZ or with *dl*922-947 24 h following siRNA transfection. Cell survival was assessed 6 h (TSZ) and 120 h (*dl*922-947) thereafter. ***p < 0.001

MLKL is the critical final mediator of necrotic cell death following multiple stimuli, including TSZ treatment. Following *dl*922-947 infection, we saw re-localisation of MLKL from the nucleus to the cell membrane (Fig. 3a), which is required for MLKL-induced necrosis^23^. Furthermore, we saw a small but significant and dose-dependent inhibition of *dl*922-947 activity following treatment with the MLKL inhibitor NSA that was similar in both TSZ-sensitive TOV21G cells and TSZ-resistant OVCAR4 (Fig. 3b). This initially suggested that MLKL may be involved in *dl*922-947-mediated cell death. However, we were unable to detect any MLKL phosphorylation following *dl*922-947 infection (Fig. 3c; see also Fig. S14). In addition, siRNA-mediated MLKL knockdown have no significant effect on virus efficacy but completely abrogated TSZ cytotoxicity (Fig. 3d). We therefore concluded that MLKL is not an absolute requirement for the death induced by adenovirus type 5.

### RIPK3 expression augments efficacy in vitro

**Fig. 4:**
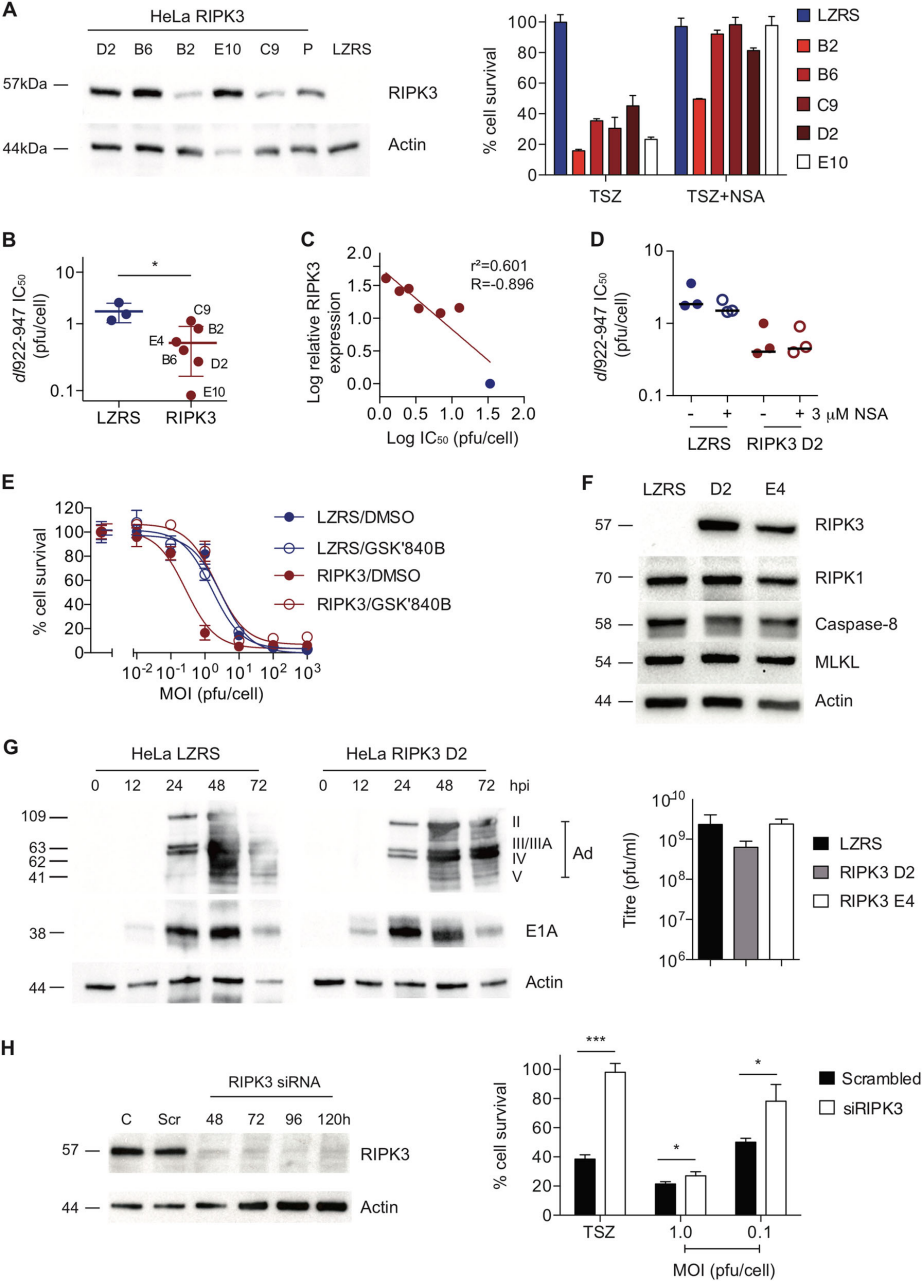
RIPK3 expression augments *dl*922-947-induced cytotoxicity in vitro **a** Following retroviral transduction, HeLa-RIPK3 single-cell clones were isolated. LZRS control clone was also isolated. RIPK3 expression (left) and sensitivity to NSA-reversible TSZ necrosis (right) were assessed. **b** HeLa-RIPK3 clones were significantly more sensitivity to *dl*922-947 than control LZRS. Each dot represents IC_50_ for each clone from a triplicate dose–response experiment. *p < 0.05. **c** Correlation between RIPK3 expression and *dl*922-947 sensitivity. Relative RIPK3 expression was quantified in HeLa-RIPK3 clones (red) and HeLa-LZRS cells (blue) from multiple immunoblots and correlated with *dl*922-947 IC_50_ values. **d** Treatment with 3 µM NSA has no effect on dl922-947 efficacy in HeLa-LZRS or HeLa-RIPK3 D2 cells. Each dot represents IC_50_ from a triplicate dose–response experiment. **e** Treatment with specific RIPK3 inhibitor GSK2791840B (10 nM) inhibited *dl*922-947 cytotoxicity in HeLa-RIPK3 (clone D2) but not HeLa-LZRS. **f** Expression of RIPK1, RIPK3, caspase-8 and MLKL was assessed in HeLa-LZRS and HeLa-RIPK3 D2 and E4 cells. **g** Expression of adenovirus proteins (left) and generation of infectious virions (right) was not increased in HeLa-RIPK3 cells compared to Hela-LZRS. Adenovirus structural protein nomenclature: II (hexon), III (penton base), IIIA, IV (fiber) and V (core). **h** RIPK3 knockdown in TOV21G cells (left) following transfection with 30 pmol siRNA. Adenovirus and TSZ cytotoxicity experiments (right). Cells were treated with TSZ or with *dl*922-947 24 h following siRNA (30 pmol) transfection. Cell survival was assessed 6 h (TSZ) and 120 h (*dl*922-947) thereafter. *p < 0.05. ***p < 0.001

We focussed next upon RIPK3, a critical kinase in both apoptosis and necrotic cell death. Following retroviral transduction, we generated a series of HeLa clones expressing RIPK3 (Fig. 4a, Fig. S6). Expression of RIPK3 increased sensitivity to TSZ, which was reversible by NSA treatment (Fig. 4a). These RIPK3 clones were also significantly more sensitive to cytotoxicity mediated by *dl*922-947 (Fig. 4b, Fig. S7) and Ad5 wild type (Fig. S8), although not by Ad11p (Fig. S9). Sensitivity to *dl*922-947 correlated significantly with the extent of RIPK3 expression (Fig. 4c) and was not affected by treatment with NSA (Fig. 4d) or RIPK1 inhibition (Fig. S10). However, sensitivity was reversed by the RIPK3 inhibitor GSK2791840B (Fig. 4e, Fig. S5). Expression of RIPK3 did not alter the expression of other core necrosis proteins (Fig. 4f) nor did it increase viral protein expression or viral replication (Fig. 4g). However, siRNA-mediated RIPK3 knockdown in TOV21G cells significantly reduced viral cytotoxicity as well as TSZ-induced death (Fig. 4h), Together, these results suggest that RIPK3 activity can augment efficacy of adenoviruses. Double siRNA for RIPK1 and RIPK3 in TOV21G cells induced significant toxicity (data not shown), so it was not possible to assess whether loss of both kinases influenced viral activity.

### RIPK3 expression improves in vivo efficacy

**Fig. 5:**
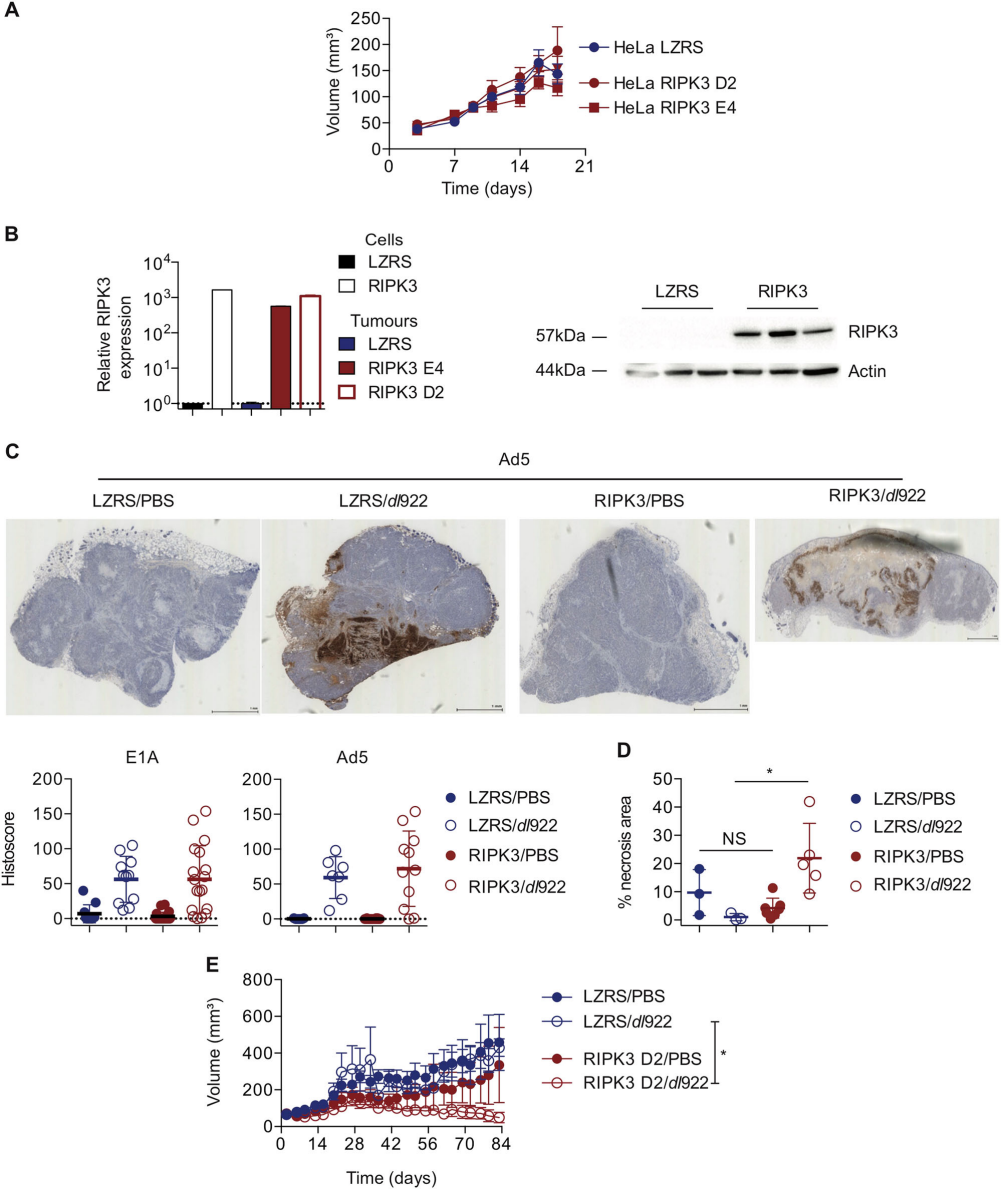
RIPK3 expression augments dl922-947-induced cytotoxicity in vivo **a** Expression of RIPK3 does not alter subcutaneous growth of HeLa tumours in female nude mice (*N* = 14 for LZRS, 28 for RIPK3). Data represent mean ± SEM. **b** HeLa-RIPK3 tumours retain expression of RIPK3 by qRT-PCR (left) and immunoblot (right). **c** HeLa-RIPK3 (clones D2 and E4) and HeLa-LZRS tumours were injected with a single intratumoural dose of *dl*922-947 (5 × 10^9^ particles) and harvested 48 h later. Expression of E1A and Ad5 proteins was assessed by quantitative IHC. **d** Necrotic area was quantified using Slidepath Tissue Image Analysis. *p < 0.05. **e** HeLa-RIPK3 (clone D2) and HeLa-LZRS tumours were grown subcutaneously in CD1 female nude mice (*n* = 12 per genotype). Once tumours reached approximately 200 mm^3^ (median 195 mm^3^ for LZRS, 185 mm^3^ for RIPK3), mice were randomly allocated to receive two doses of intratumoural *dl*922-947 (5 × 10^9^ particles) or PBS, 14 days apart. Tumours were measured twice weekly by callipers. Error bars represent SEM. *p < 0.05

We then evaluated the effect of RIPK3 expression in vivo. We first confirmed that RIPK3 expression did not alter growth of subcutaneous xenografts from two HeLa RIPK3 clones, D2 and E4 (Fig. 5a), and that RIPK3 expression was maintained in vivo (Fig. 5b). Expression of E1A and adenovirus structural proteins was assessed using quantitative immunohistochemistry (IHC) in control (HeLa LZRS) and RIPK3-expressing tumours following a single intratumoural injection of *dl*922-947. As with the in vitro data, there was no difference in viral protein expression (Fig. 5c), but we did detect a significant increase in total necrotic area in RIPK3-expressing tumours following *dl*922-947 injection compared to HeLa LZRS (Fig. 5d). In a therapeutic experiment using the HeLa-RIPK3 D2 clone, RIPK3 expression significantly enhanced the activity of intratumoural *dl*922-947 (Fig. 5e, Fig. S11), with complete elimination of 3/6 HeLa-RIPK3 D2 tumours compared to 0/6 HeLa-LZRS tumours.

### Caspase-8 inhibition augments viral cytotoxicity that is reversed by RIPK3 inhibition and MLKL knockout

**Fig. 6:**
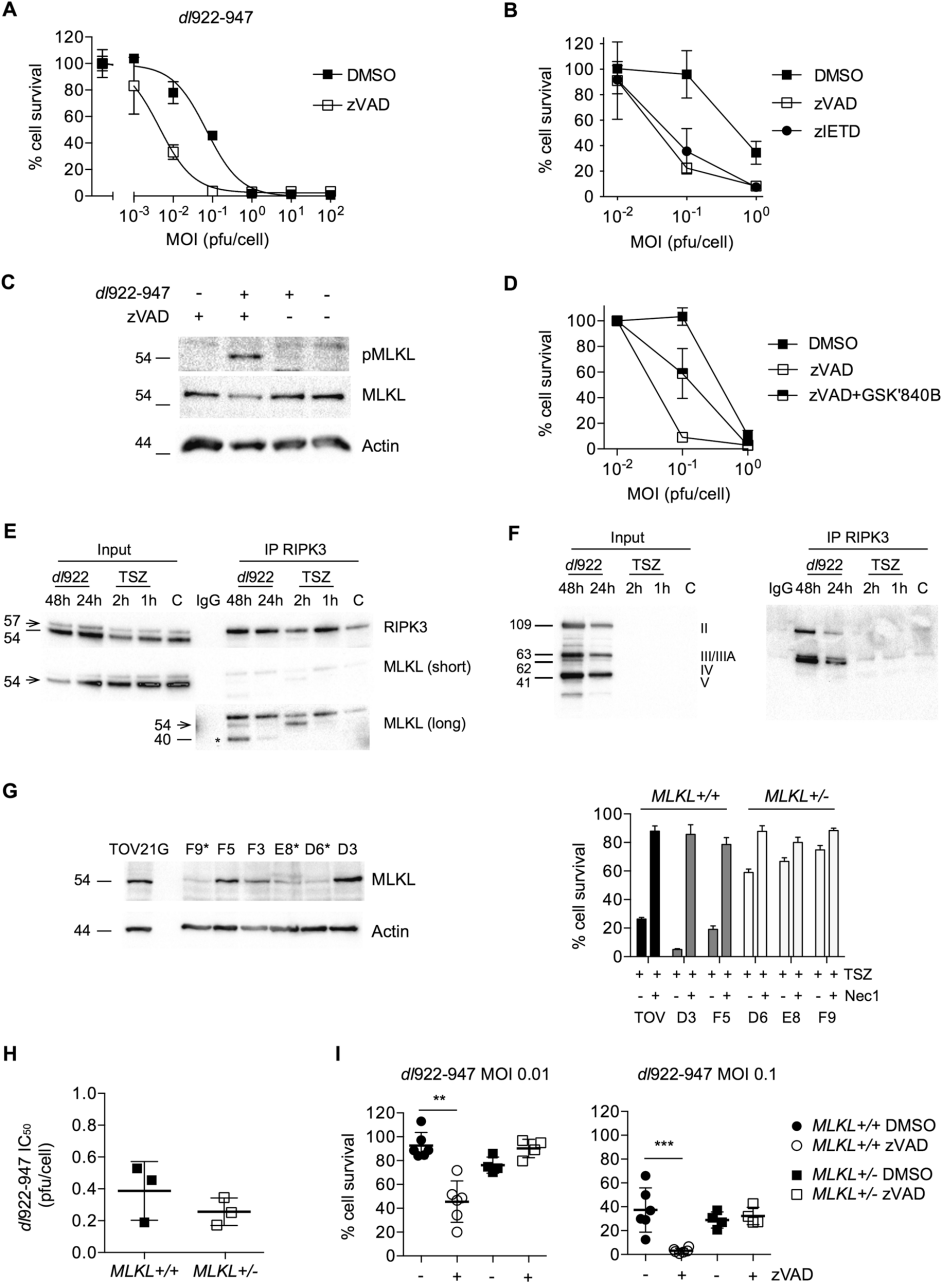
Caspase-8 inhibition augments viral cytotoxicity that is reversed by RIPK3 inhibition and MLKL knockout **a** TOV21G cells were infected with *dl*922-947 (MOI 0.001–100 pfu/cell). zVAD.fmk (25 µM) or DMSO was added 2 h postinfection. Cell survival was assessed 120 h postinfection. **b** TOV21G cells were infected with *dl*922-947 (MOI 0.01–1 pfu/cell). zVAD.fmk (25 µM), zIETD.fmk (25 µM) or DMSO was added 2 h postinfection. Cell survival was assessed 120 h postinfection. **c** MLKL phosphorylation was assessed in TOV21G following treatment with *dl*922-947 (MOI 1, 72 h) and/or zVAD.fmk (25 µM, 72 h) by immunoblot. **d** TOV21G cells were infected with *dl*922-947 (MOI 0.001–1). zVAD.fmk (25 µM) with or without GSK2791840B (10 µM) was added 2 h later. Cell survival was assessed 120 h postinfection by MTT assay. **e**, **f** HeLa-RIPK3 D2 cells were infected with *dl*922-947 (MOI 1) for up to 48 h or treated with TSZ for up to 2 h. RIPK3 was immunoprecipitated as described in Materials and Methods. RIPK3 and MLKL **e** and adenovirus proteins **f** were assessed in lysate and precipitate by immunoblot. Arrow in **e** represents MLKL band at 54 Kda. Asterisks indicates non-specific band. **g** Single-cell TOV21G clones were isolated following CRISPR/Cas9-mediated *MLKL* exon 5 editing. MLKL expression was assessed by immunoblot (left). Clones marked asterisk (F9, E8, D6) had reduced MLKL expression and demonstrated mono-allelic deletions in exon 5 by sequencing. *MLKL*^*+/*^^−^ clones had reduced sensitivity to TSZ-induced necrosis (right). **h** No difference in *dl*922-947 sensitivity in *MLKL*^*+/*^^−^ clones compared to *MLKL*^*+/+*^. Each dot represents IC_50_ for one clone from a triplicate dose–response experiment. **i** Loss of MLKL reverses zVAD.fmk-induced enhancement of *dl*922-947 cytotoxicity. Each point represents a single triplicate experiment at each MOI. **p < 0.01. ***p < 0.001. Concentration of zVAD.fmk was 25 µM

Finally, we sought to explain how RIPK3 influences adenovirus activity. Previously, we showed that zVAD.fmk, the pan-caspase inhibitor, does not reverse *dl*922-947 efficacy in multiple ovarian cancer cells^5^, including OVCAR4, which we re-confirmed here (Fig. S12). However, we observed here that addition of 25 µM zVAD.fmk consistently and significantly increased the activity of *dl*922-947 (Fig. 6a) and Ad5 (Fig. S13) in TOV21G. Moreover, this effect was recapitulated following treatment of TOV21G with the caspase-8-directed inhibitor zIETD.fmk (25 µM) (Fig. 6b). There was clear evidence of MLKL phosphorylation 72 h following *dl*922-947 infection in the presence of zVAD.fmk (Fig. 6c) but not at 24 or 48 h (Fig. S14), and the effect of zVAD.fmk was partially rescued by the RIPK3 inhibitor GSK2791840B (Fig. 6d). To explore further, we performed RIPK3 co-immunoprecipitation and demonstrated an interaction between RIPK3 and MLKL following *dl*922-947 infection (Fig. 6e, Fig. S15) as well as an interaction between RIPK3 and adenovirus proteins (Fig. 6f). We then used CRISPR/Cas9 gene editing to knock out MLKL in TOV21G cells. We were unable to isolate any clones with bi-allelic mutations in *MLKL* but did identify three heterozygote clones (Supplementary data) with reduced MLKL expression by immunoblot and reduced sensitivity to TSZ-induced necrosis compared to both parental TOV21G cells and clones with no *MLKL* mutations (Fig. 6g). The partial loss of MLKL did not increase sensitivity to *dl*922-947 significantly (Fig. 6h), in keeping with siRNA experiments (Fig. 3d), but completely reversed the zVAD.fmk-mediated increase in *dl*922-947 efficacy (Fig. 6i).

## Discussion

Evasion of cell death is a hallmark of cancer^24^, and all anticancer therapies must circumvent these death-resistance mechanisms to be effective. Oncolytic viruses are a novel form of therapy that can both induce direct cytotoxicity in infected cells and establish adaptive antitumour immune responses^25^. However, the mode of death induced by adenovirus remains unclear.

We previously showed that E1A CR2-deleted serotype 5 adenoviruses induce a form of programmed death that was not apoptotic but had features to suggest a type of necrosis^5^. However, there have been few attempts to interrogate adenovirus-induced cell death since the recent elucidation of programmed necrosis pathways. Our results here indicate that the group C adenoviruses *dl*922-947 and Ad5 wild type induce a mode of cell death that morphologically appears necrotic with associated membrane rupture. We show that these viruses do not require the necrotic kinase RIPK1 and that blockade of TNF-α does not inhibit viral activity. Although NSA treatment partially inhibited cytotoxicity in a dose-dependent manner in TOV21G and OVCAR4 cells, suggesting that MLKL might play a role, siRNA experiments were negative (Fig. 3d), NSA treatment had no effect in the HeLa-RIPK3 clones and there was no MLKL phosphorylation at 24 and 48 h hours postinfection (Fig. S14), implying that the NSA effect represented off-target activity. By contrast, RIPK3 does play a role, with reduction in cytotoxicity upon siRNA-mediated knockdown in TOV21G cells and increased cell death both in vitro and in vivo upon RIPK3 overexpression in HeLa. Moreover, we also show that inhibition of caspases, specifically caspase-8, significantly augments activity in necrosis-competent TOV21G cells, in a manner that is RIPK3 and MLKL dependent.

The first conclusion of these results is that group C adenovirus-induced death differs from the classical pathway of necroptosis induced by TSZ (TNF-α, Smac mimetic and zVAD.fmk) and does not absolutely require the presence of RIPK1, RIPK3 and MLKL; HeLa cells do not express RIPK3, while OVCAR4 express low levels of both RIPK3 and MLKL, and both are resistant to TSZ treatment. Yet both still undergo cytotoxicity following adenovirus infection (Fig. 1a–c). Nonetheless, TOV21G cells, which are intrinsically TSZ sensitive, are the most susceptible to adenovirus, as we have shown previously^3,22^, and siRNA-mediated RIPK3 knockdown partially enhanced cytotoxicity in these cells. Furthermore, HeLa-RIPK3 cells, which are rendered TSZ-sensitive through RIPK3 expression, had increased adenovirus sensitivity, with a direct correlation between extent of RIPK3 expression and sensitivity. Together, these results suggest a link between virus activity and programmed necrosis.

The second conclusion is that adenovirus-induced death parallels that induced by other DNA viruses, including murine cytomegalovirus (mCMV). In mCMV infection, RIPK1 is also redundant, and caspase-8 acts to suppress RIPK3-mediated death^18^. In addition, the mCMV M45 gene encodes viral inhibitor of RIP activation, which contains a RHIM domain and blocks RIPK3-dependent death^18^. Here co-immunoprecipitation experiments demonstrate that a RIPK3- and MLKL-containing complex forms following *dl*922-947 infection and that MLKL phosphorylation (and hence activation) is evident upon caspase inhibition. Thus, in TOV21G cells, caspase-8 acts to suppress virus-induced necrosis. Our data are also intriguing in that, in the absence of caspase inhibition, knockdown of RIPK3, but not MLKL, partially reduces cytotoxicity (Fig. 4f), suggesting that there may be other death effector proteins that are activated by RIPK3 in the presence of adenovirus infection. We hypothesise that there are critical interactions between adenovirus proteins and RIPK3 that inhibit RIPK3 function and thus prevent MLKL phosphorylation in the absence of caspase-8 inhibition. However, unlike mCMV and HSV-1, there are no published reports of RHIM domain-containing adenovirus proteins. Co-immunoprecipitation here did suggest direct interaction between RIPK3 and adenovirus capsid proteins, but further analysis will be required to establish the specificity and significance of these interactions, as well as to identify other adenovirus proteins that might bind to RIPK3.

It is known that adenovirus encodes proteins that can specifically inhibit mitochondrial apoptosis, especially E1B 19K, a Bcl2 homologue that blocks Bax homo/heterodimerisation^26,27^. Other adenovirus proteins involved in cell death include E3 11.6K (adenovirus death protein, ADP) and E4orf4. ADP is expressed late following infection and promotes lysis of infected cells^28,29^ and may be a critical regulator of lytic versus latent infection in lymphocytes^30^. However, there are no publications to suggest that ADP-mediated lysis might represent a necrotic process nor that ADP interacts with RIPK3 or MLKL. E4orf4, when expressed alone, can induce p53-independent, caspase-independent cell death^31,32^, but again, there is no evidence to support an interaction with any necrotic proteins, nor indeed to support a role for E4orf4 in cell death during productive adenovirus infection^33^.

Dyer et al. have recently investigated the group B adenovirus enadenotucirev (EnAd) in A549 cells^34^ and suggested that, rather than a necrotic process, cytotoxicity resembled ischaemic oncosis, a form of death marked by ATP loss, a rise in intracellular calcium and loss of membrane integrity with release of inflammatory mediators^35^. Some of the changes seen following EnAd infection were replicated following Ad11p and Ad5 infection, although they were less marked. Our data do not necessarily contradict these results, as loss of ATP and membrane integrity are also observed in necrosis^36^, including that induced by vaccinia, as we have previously noted^16^.

Other viruses also interact with RIPK3, including influenza A virus (IAV). IAV infection activates RIPK3, which then triggers parallel pathways of cell death, including MLKL-dependent necrosis and FADD-dependent apoptosis^19^. The key sensor for RIPK3 activation is DAI, which recognises IAV RNA^37^. Thus other host proteins beyond the core necrotic machinery may be responsible for directing cell death. It is noticeable that the enhancement of *dl*922-947-induced cytotoxicity seen in the presence of zVAD.fmk and zIETD.fmk in TOV21G cells was not recapitulated in HeLa-RIPK3 cells (data not shown) despite expression of caspase-8 in these cells, suggesting the presence of specific pathways within TOV21G cells responsible for these observations. Other potential cellular pathways to trigger RIPK3 activation include TLR signalling. Inhibition of caspase-8 in the presence of activated TLR signalling, including TLR3 and TLR4, results in RIPK3-dependent necrosis that requires TRIF or MyD88 signalling^12^. It is certainly known that adenovirus can activate multiple TLR signalling pathways, including TLR4^38^. Recent data also suggest that human adenovirus uptake can induce very rapid necrosis of liver macrophages that is independent of RIPK3 activity, but relies upon the transcription factor IRF3 (interferon-regulatory factor 3), and is triggered by viral entry into the cytosol^39^. However, the kinetics of liver macrophage death, which was observed within 5–10 min of intravenous injection of human adenovirus into wild-type C57Bl/6 mice, are dramatically different from that seen following infection of human epithelial cells and occuring prior to any viral gene expression. One difficulty of investigating adenovirus-induced death in vivo is the species specificity of human adenovirus. We have previously identified failure of translation of late human adenovirus mRNA in murine cells as a key factor in that specificity^40^, which precludes the use of transgenic murine models as tools to investigate the role of specific host genes either in whole animals of murine embryonic fibroblasts. However, a valuable lesson from the investigation of HSV-1 is that host species is critical—the ICP6/RIPK3 interaction has polar opposite effects in murine and human cells^20,21^—implying strong evolutionary pressure in natural hosts to protect against from virus infection^41^.

**Fig. 7:**
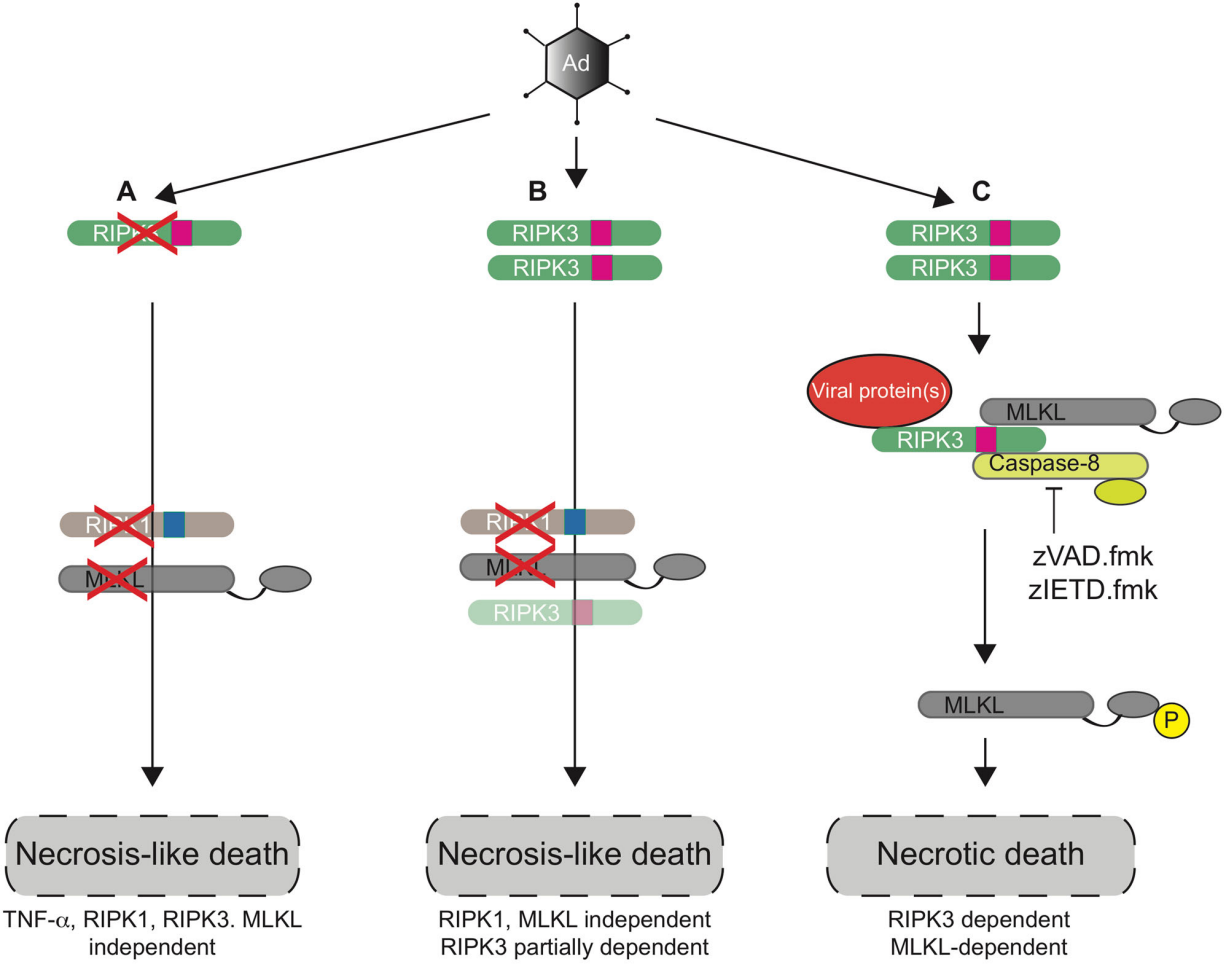
Schematic representation of adenovirus-induced cell death **a** In cells lacking RIPK3 (or expressing low levels of RIPK3), adenovirus infection induces cell death that resembles necrosis but is TNF-α, RIPK1, RIPK3 and MLKL independent and is not apoptotic. **b** In the presence of RIPK3, either endogenous or via overexpression, cells undergo RIPK1- and MLKL-independent cell death that is partially RIPK3 dependent. **c** In TOV21G cells, adenovirus infection leads to formation of a complex containing RIPK3, MLKL and viral protein(s). In the presence of either zVAD.fmk or zIETD.fmk, inhibition of caspase-8 leads to MLKL phosphorylation and necrotic cell death that is both RIPK3 and MLKL dependent

In summary (Fig. 7), we show here that group C adenovirus-induced death has some hallmarks of necrosis that differs from the classical TSZ-induced death and is specifically independent of TNF-α and RIPK1. In the presence of caspase-8 inhibition, death can proceed in a RIPK3- and MLKL-dependent pathway. Future work will be required to identify whether specific adenovirus proteins regulate this effect and the key host signalling pathways involved.

## Materials and methods

### Cell lines, viruses and viability assays

HeLa and 293T cells were obtained from Cancer Research UK Cell Services (Clare Hall, UK), OVCAR4 from National Cancer Institute (Frederick, MA, USA) and TOV21G from Professor Fran Balkwill (Barts Cancer Institute, London, UK). The Phoenix-AMPHO cells were kindly provided by Stephen Tait’s laboratory. Cells were cultured in Dulbecco’s Modified Eagle Medium with 10% heat-inactivated foetal bovine serum, 2 mM L-Glutamine, 100 units/ml penicillin and 100 μg/ml streptomycin. All cell lines were verified by STR profiling (Promega GenePrint 10) at the CRUK Beatson Institute. Cells were tested for mycoplasma every second week. Cell survival was assessed by MTT assay^42^ and by flow cytometry following staining with Zombie Violet membrane-impermeable fluorescent dye (Biolegend, London, UK).

The Ad5 vector, *dl*922-947, has been described previously. It contains a 24-bp deletion in E1A CR2 as well as a 745-bp deletion in E3B. The Adenovirus Death Protein (E3 11.6K) is retained^43^. Adenoviruses type 5, 11 and 35 were obtained from Dr H Wang, Barts Cancer Institute, London, UK. Virus replication was assessed by TCID50 assay as previously^1^.

HeLa RIPK3 and control (LZRS) cells were generated by retrovirus transduction of HeLa following transient transfection of the plasmids pLZRS-RIPK3/pLZRS-control^44,45^ into Phoenix-AMPHO cells. Following selection in zeocin, single-cell clones were isolated by dilution cloning.

### Necrosis induction and inhibition

Cells treated with TSZ (TNF-α (20 ng/ml), Smac-mimetic (1 μM LCL-161) zVAD.fmk (25 µM)) for up to 72 h. Necrostatin-1 and NSA were obtained from Enzo Life Sciences, Switzerland and Calbiochem, USA, respectively. Specific RIPK1 (GSK3002962A, GSK3002963A) and RIPK3 (GSK2791840B) inhibitors were kind gifts from Dr Peter Gough, GlaxoSmithKline (Collegeville, PA, USA).

### Immunoblotting and co-immunoprecipitation

Twenty micrograms of total protein was electrophoresed at 140 V for 1 h, transferred onto nitrocellulose and blocked in 5% non-fat milk. A full list of antibodies is given in Supplementary Material. Membranes were exposed on a Chemi-doc MP (Biorad) with ECL (GE Healthcare, UK). RIPK3 expression was quantified using ImageJ (NIH, v1.46r).

For co-immunoprecipitation, cells were lysed in Nonidet P-40 buffer (10 mM Tris pH 8.0, 150 mM NaCl, 1% Nonidet P-40) with protease and phosphatase inhibitor cocktail 2 (Sigma-Aldrich, UK). One milligram of total protein was incubated overnight at 4° C with anti RIPK3 Ab (Santa-Cruz Sc374639, 1:50). Samples were incubated for 2 h with 15 μl Dynabead Pan Mouse IgG magnetic beads (Invitrogen, UK) and washed three times in ice-cold Nonidet P-40 buffer. To pull down the desired complex, 16 μl of water and 4 μl of 5x Laemmli buffer were added to the beads, vortexed and denatured at 95° C for 5 min. Beads were removed and the samples were electrophoresed as above.

### Electron microscopy

Cells grown on Thermanox coverslips were washed in phosphate-buffered saline (PBS) before fixing in 1.5% Glutaraldehyde/0.1 M Sodium Cacodylate buffer for 1 h at 4° C and then washed three times for 5 min each in 0.1 M Sodium Cacodylate buffer rinse (2% sucrose). Samples were postfixed in 1% Osmium Tetroxide/0.1 M Sodium Cacodylate buffer for 1 h and then washed for 3 × 10 min in distilled water followed by en block staining in 0.5% aqueous Uranyl Acetate for 1 h in the dark and washed again twice for 1 min with dH_2_O. Samples were dehydrated through a graded ethanol series (30, 50, 70, 90%) for 10 min followed by 100% ethanol 4 × 5min, followed with Propylene Oxide 3 × 5min and then 1:1 Propylene Oxide: Araldite/Epon resin (TAAB 812) overnight. Samples were then put into fresh pure Epon/Araldite resin, embedded in flat bed moulds and polymerized for 48 h at 60° C. Ultrathin sections (60–70 nm) were produced using a LEICA Ultracut UCT (Leica Microsystems, UK) and Diatome diamond knife (Diatome, USA) at an angle of 6 degrees. Sample sections were picked up on 100 mesh formvar-coated copper grids and then contrast stained with 2% Methanolic Uranyl Acetate for 5 min followed by Reynolds Lead Citrate for 5 min. Samples were viewed on a FEI Tecnai T20 (Zeiss, UK) at an accelerating voltage of 200 kV, and images were captured using the GATAM Digital Imaging system.

### Confocal microscopy

Cells were seeded on coverslips, infected with *dl*922-947 (MOI 1, 48 h), permeabilised with 0.2% Triton X-100 (Sigma) in PBS for 1 min and then fixed in 4% paraformaldehyde for 10 min. Cells were stained with anti-MLKL antibody (Millipore, Watford, UK) and co-stained with anti-phalloidin (Santa Cruz, CA, USA) antibody for 1 h at room temperature. Secondary antibodies were incubated for 1 h in the dark at room temperature. Cells were co-stained with 4,6-diamidino-2-phenylindole. Coverslips were mounted on slides and images were captured using a Zeiss 710 confocal microscope.

### CRISPR/Cas9 gene editing

Two open-access software programs, CHOPCHOP (https://chopchop.rc.fas.harvard.edu/) and CRISPR design (http://crispr.mit.edu/), were used to design guide RNAs (gRNA) targeted to *MLKL* exon 5. Three guides were designed, although only one guide, which targeted the RIPK3 phosphothreonine target site of *MLKL* (nt 25408-25437 inclusive), yielded knockout clones. Annealed oligonucleotides were ligated into BbsI-linearised pSpCas9(BB)-2A-Puro (PX459 v2—Addgene no. 62988^46^, a gift from Feng Zhang via Addgene). All plasmids were sequenced to confirm successful ligation.

TOV21G cells (4 × 10^5^) were plated overnight in antibiotic-free medium and transfected with 4 µg PX459 using Lipofectamine 2000, selected under puromycin (2.5 µg/ml) for 48 h and plated onto 96-well plates (10 cells/ml). Single-cell colonies were expanded for DNA extraction, protein extraction and cryopreservation.

PCR primers spanning potential sites of deletion were designed (Forward 5ʹ-ACAATCCCTGCCCTTTACTCC-3ʹ, Reverse 5ʹ-GAGTTTAGGTGGTCCTTGGAGG-3ʹ). Clones with large PCR insertion/deletions were selected for subsequent analysis. Remaining clones were screened using the Surveyor Nuclease Assay (Integrated DNA Technology). Mutations were confirmed by Sanger sequencing. All sequence alignment was performed using MAFFT version 7 (http://mafft.cbrc.jp/).

### In vivo experiments

All animal experiments were performed in the Cancer Research UK Beatson Institute Biological Services Unit (registered facility 60/2607) under appropriate UK Home Office personal and project licence (70/8645) authority. All experiments adhered to NCRI Guidelines on the use of animals in medical research^47^. Animals were allocated treatment randomly by cage. All injections and tumour measurements, as well as all decisions about animal welfare, were taken by BSU staff and D.A. to prevent bias.

Female CD1 nu/nu mice were injected subcutaneously with 5 × 10^6^ cells in 100 μl of PBS in groups of 6. Tumours were measured three times a week, and volume was calculated using the equation (*L* × *W*^2^)/2, where *L* = longest diameter and *W* = perpendicular width. Once tumours reached approximately 200 mm^3^, each group received intratumoural doses of *dl*922-947 (5 × 10^9^ particles in 30 µl) or PBS (30 µl). For tumour IHC and immunoblot experiments (Fig. 5b – d), both HeLa RIPK3 D2 and HeLa RIPK3 E4 tumours were used. Tumours were harvested 48 h after single injection of *dl*922-947 (5 × 10^9^ particles) or PBS and cut into two equal parts. One was snap frozen and the other fixed overnight in 10% paraformaldehyde before processing. Slides were stained for the expression of E1A (rabbit anti Ad2/5 E1A, sc-430, 1:50, Santa-Cruz, CA) or adenovirus structural proteins (goat anti-adenovirus, ab36851, 1:400, Abcam, UK). Stained slides were digitised (Hamamatsu NanoZoomer NDP, Hamamatsu Photonics, Welwyn Garden City, UK) and viewed using Slidepath Digital Image Hub V4.0.7 (Leica Microsystems, Milton Keynes, UK). Areas of tumour were identified and scored using Slidepath Tissue Image Analysis and histoscores were generated by multiplying intensity of cellular staining within marked areas (range 0–3) by the percentage of cells with positive staining (range 0–100), with a maximum histoscore of 300. Tumour necrotic area was calculated as the percentage of acellular/anuclear area per tumour divided by total tumour area using Slidepath Tissue Image Analysis.

In the therapeutic experiment (Fig. 5e), HeLa RIPK3 D2 cells were utilised and two doses of virus were administered, 14 days apart (days 23 and 37 for LZRS tumours; days 27 and 41 for RIPK3). Mice were killed when tumours reached clinical end point (maximum length 15 mm or tumour ulceration).

### Statistics

All data points show mean ± SD unless otherwise stated. All statistical calculations were performed using Prism (v6, GraphPad, CA). All comparisons utilise unpaired *t-*test unless otherwise stated. *p* < 0.05 was considered significant.

## Electronic supplementary material

Supplementary Figures legends

Supplementary Figures 1 and 2

Supplementary Figures 3 - 10

Supplementary Figures 11 - 15

Supplementary sequence alignment
