# Supplementary material for: RIPK3 promotes adenovirus type 5 activity
Source: Cell Death Dis. 2017 Dec 13;8(12):3206. doi: 10.1038/s41419-017-0110-8 (PMC5870599; doi:10.1038/s41419-017-0110-8)
Supplement: Supplementary file 1 — Supplementary Figures legends [file 41419_2017_110_MOESM1_ESM.docx]

# Supplementary material

## Supplementary Figure Legends

Figure S1. Transmission electron microscopy of TOV21G (a) OVCAR4 cells (b) infected with *dl*922-947 (MOI 1 and 10 respectively) for 48h. TOV21G image is composite of four individual EM micrographs (see Fig. 1C). Enlarged OVCAR4 image (right) shows intra-nuclear tessellations of capsid proteins (C) as well as whole virions (arrows).

Figure S2. Assessment of membrane integrity following *dl*922-947 infection. TOV21G and OVCAR4 cells were infected with *dl*922-947 (MOI 1 and 10 respectively) for up to 72h. Cells were assessed by flow cytometry following staining with Zombie Violet (Biolegends, UK), a membrane impermeable amine-reactive fluorescent dye. Upper panel shows loss of membrane integrity in TOV21G cells up to 72 post-infection. Lower panel shows multiple assessments 48h post-infection in both cell lines.

Figure S3. E1A and caspase-8 expression was assessed by immunoblot up to 72h hours post-infection (hpi) of TOV21G with *dl*922-947 (MOI 1).

Figure S4. Necrostatin-1 (10 µM) has no effect on *dl*922-947 efficacy. Each dot represents IC50 for single triplicate experiment.

Figure S5. Effect of specific RIPK1 inhibitors (RIPK1i) and RIPK3 inhibitors (RIPK3i) in TSZ-induced necrosis in TOV21G cells. Cell survival was assessed by MTT assay following 6h treatment.

Figure S6. Expression of RIPK3 in HeLa-RIPK3 clones. P = HeLa-RIPK3 pool. LZRS = empty-vector control.

Figure S7. Representative *dl*922-947 dose response curves in HeLa-RIPK3 clones. Survival was assessed 120h post-infection.

Figure S8. Ad5 wild-type dose responses in HeLa-LZRS and HeLa-RIPK3 (clone D2) cells. Survival was assessed 120h post-infection.

Figure S9. Ad11p wild-type dose responses in HeLa-LZRS and HeLa-RIPK3 (clone D2) cells. Survival was assessed 120h post-infection.

Figure S10. HeLa-RIPK3 D2 cells were infected with *dl*922-947. RIPK3 inhibitors or DMSO were added two hours post-infection. Cell survival was assessed 120h post-infection.

Figure S11. Individual tumour volume data from Fig. 5E for HeLa-LZRS and HeLa-RIPK3 tumours following two intra-tumour injections of *dl*922-947

Figure S12. zVAD.fmk (25 µM) has no effect on *dl*922-947 efficacy in OVCAR4 cells.

Figure S13. zVAD.fmk (25 µM) augments Ad5 WT efficacy in TOV21G cells.

Figure S14. MLKL phosphorylation was assessed in TOV21G following treatment with *dl*922-947 (MOI 1) and/or zVAD.fmk (25 µM) for 24h and 48 hours by immunoblot. See Fig. 6C for 72h blot.

Figure S15. RIPK3 immunoblot following RIPK3 immunoprecipitation in HeLa-RIPK3 D2 and HeLa-LZRS cells. IN = input; IP = immunoprecipitate; SN = supernatant.

##

## Supplementary sequence alignment

Alignment of Sanger sequencing for three TOV21G clones containing mono-allelic *MLKL* alterations. The comparator sequence was human *MLKL* nt 25310 - 25632. Clones D6 and F9 both contain a 104b.p. insertion at n.t. 25424; clone E8 contains a 10 b.p. deletion between nt 70-79 inclusive.

## Supplementary tables

Table S1: Primary Antibodies used for Western Blotting.

| **Primary Antibodies** | **Catalogue number** | **Condition** | **Supplier** |
| --- | --- | --- | --- |
| Adenovirus-2/5 E1A | sc-430 | 1:1000 in 5% BSA TBST | Santa Cruz Biotechnology, USA |
| Anti-Adenovirus | ab36851 | 1:500 in 3% BSA TBST | Abcam, UK |
| Caspase 8 | 554002 | 1:1000 in 5% BSA TBST | BD Pharmingen, UK |
| Caspase 8 | 4790 | 1:300 in 5% BSA TBST | Cell Signaling, USA |
| FADD | 610399 | 1:1000 in 5% BSA TBST | BD Pharmingen, UK |
| GAPDH | ab9485 | 1:1000 in 3% BSA TBST | Abcam, UK |
| MLKL | MABC604 | 1:1000 in 3% BSA TBST | Millipore, UK |
| MLKL | ab184718 | 1:1000 in 3% BSA TBST | Abcam, UK |
| Phospho-MLKL | 91689 | 1:1000 in 5% BSA TBST | Cell Signaling, USA |
| RIPK1 | 3493 | 1:1000 in 5% BSA TBST | Cell Signaling, USA |
| RIPK3 | NBP2-24588 | 1:1000 in 3% BSA TBST | Novus Biologicals, UK |
| Β-Actin | ab6276 | 1:1000 in 3% BSA TBST | Abcam, UK |

Table S2: Secondary Antibodies used for Western Blotting.

| **Secondary Antibodies** | **Catalogue number** | **Condition** | **Supplier** |
| --- | --- | --- | --- |
| Goat Anti-Mouse heavy chain | ab97240 | 1:1000 in 2% BSA TBST | Abcam, UK |
| Rabbit Anti-Mouse | P026002-2 | 1:1000 in 2% BSA TBST | Dako, USA |
| Goat Anti-Rabbit | P044801-2 | 1:2000 in 2% BSA TBST | Dako, USA |
| Rabbit Anti-Goat | P044901-2 | 1:1000 in 2% BSA TBST | Dako, USA |
| Rabbit Anti-Rat | P0450 | 1:1000 in 2% BSA TBST | Dako, USA |
| Rat Anti-Mouse kappa light chain | ab99632 | 1:2000 in 3% BSA TBST | Abcam, UK |
