# Supplementary figures and images for: RIPK3 promotes adenovirus type 5 activity
Source: Cell Death Dis. 2017 Dec 13;8(12):3206. doi: 10.1038/s41419-017-0110-8 (PMC5870599; doi:10.1038/s41419-017-0110-8)

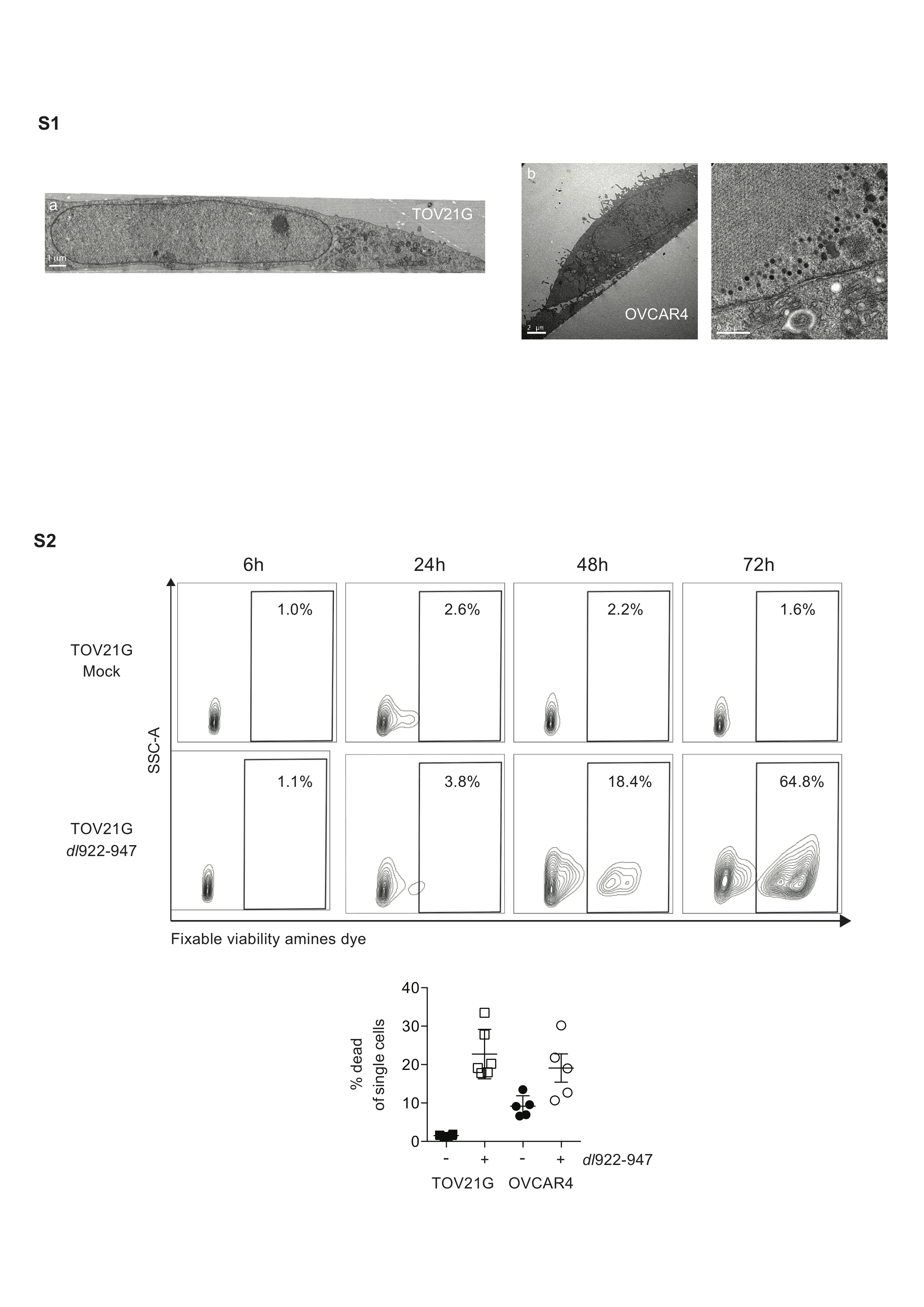

Supplement: Supplementary file 2 — Supplementary Figures 1 and 2 [file 41419_2017_110_MOESM2_ESM.jpg]

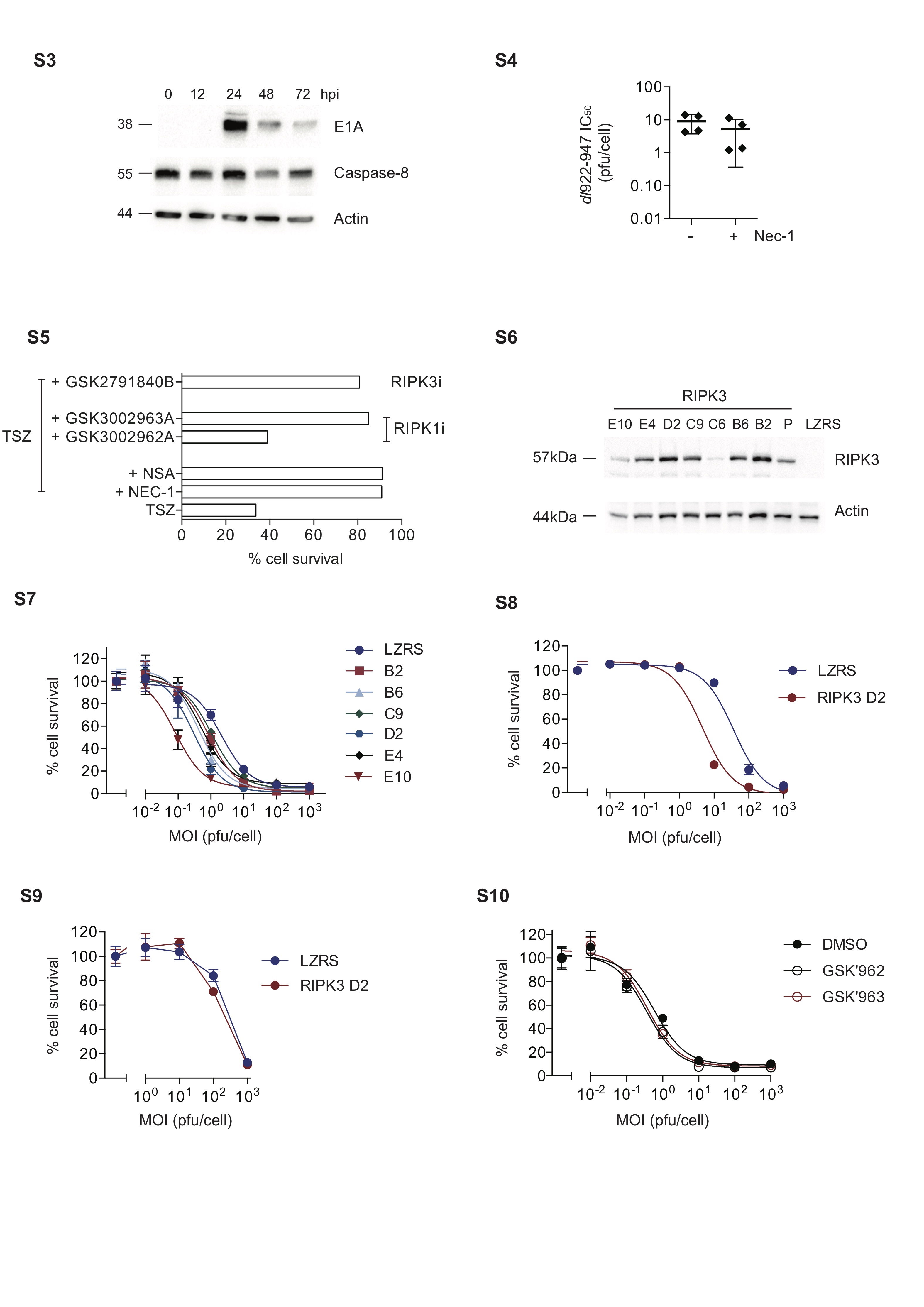

Supplement: Supplementary file 3 — Supplementary Figures 3 - 10 [file 41419_2017_110_MOESM3_ESM.jpg]

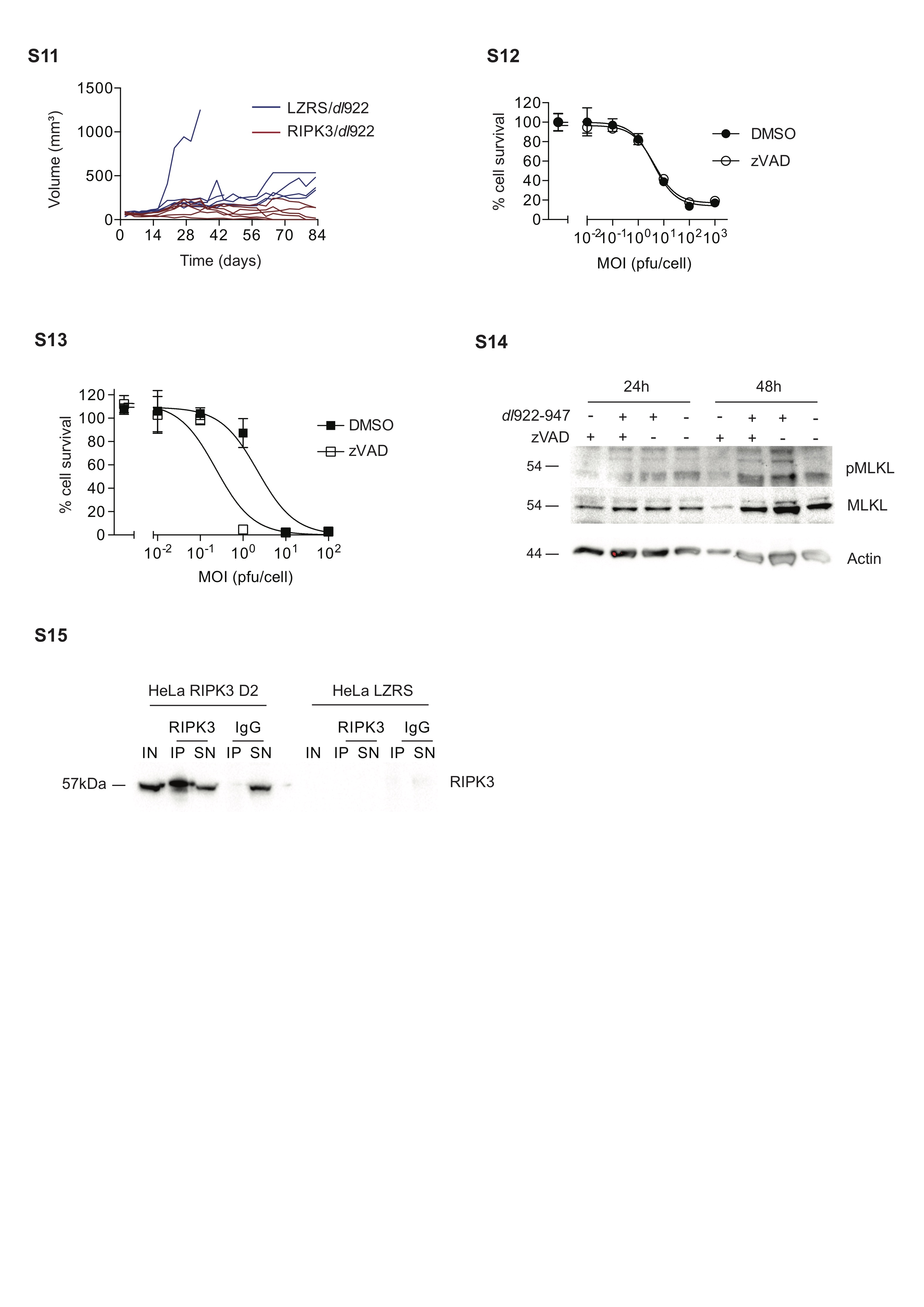

Supplement: Supplementary file 4 — Supplementary Figures 11 - 15 [file 41419_2017_110_MOESM4_ESM.jpg]
