# Supplementary material for: RIPK3 promotes adenovirus type 5 activity
Source: Cell Death Dis. 2017 Dec 13;8(12):3206. doi: 10.1038/s41419-017-0110-8 (PMC5870599; doi:10.1038/s41419-017-0110-8)
Supplement: Supplementary file 5 — Supplementary sequence alignment [file 41419_2017_110_MOESM5_ESM.rtf]

D6  1          11         21         31         41        50          |          |          |          |          |        |    ---------- ---------- ---------- -GTAGCAACA ATCCCTGCCC   19 MLKL (25310-25632)      AAGGTAAAAG ACAATTCCCT GGCAATTGTG TGTAGCAACA ATCCCTGCCC  342 D6_24     AAGGTAAAAG ACAATTCCCT GGCAATTGTG TGTAGCAACA ATCCCTGCCC  350 D6_19     TTTACTCCTC TACCCAAGAT CTTGCTTGTT TCTTTCTAAG TTGCCTCTCT   69 MLKL (25310-25632)      TTTACTCCTC TACCCAAGAT CTTGCTTGTT TCTTTCTAAG TTGCCTCTCT  392 D6_24     TTTACTCCTC TACCCAAGAT CTTGCTTGTT TCTTTCTAAG TTGCCTCTCT  400 D6_19     ATCTAGCTTG CAGGATTTGA GTTGAGGAAA ACACAGACTT CCATG-----  114 MLKL (25310-25632)       ATCTAGCTTG CAGGATTTGA GTTGAGGAAA ACACAGACTT CCATG-----  437 D6_24     ATCTAGCTTG CAGGATTTGA GTTGAGGAAA ACACAGACTT CCATGAGAGT  450 D6_19     ---------- ---------- ---------- ---------- ----------  114 MLKL (25310-25632)       ---------- ---------- ---------- ---------- ----------  437 D6_24     TGAGTGTTGT TCCAGTTTGG AACAAGAGTC CACTATTAAA GAACGTGGAC  500 D6_19     ---------- ---------- ---------- ---------- ---------A  115 MLKL (25310-25632)      ---------- ---------- ---------- ---------- ---------A  438 D6_24     TCCAACGTCA AAGGGCGAAA AACCGTCTAT CAGGGTGATG GTTCACGTAA  550 D6_19     GTTTGGGAAC TACGAGAGAA AAGACAGACA GAGTCAAATC TACAGCATAT  165 MLKL (25310-25632)      GTTTGGGAAC TACGAGAGAA AAGACAGACA GAGTCAAATC TACAGCATAT  488 D6_24     GTTTGGGAAC TACGAGAGAA AAGACAGACA GAGTCAAATC TACAGCATAT  600 D6_19     CTCTCACCTC AGGAACTGGA AGATGTATTT TATCAATATG ATGTAAAGTC  215 MLKL (25310-25632)     CTCTCACCTC AGGAACTGGA AGATGTATTT TATCAATATG ATGTAAAGTC  538 D6_24     CTCTCACCTC AGGAACTGGA AGATGTATTT TATCAATATG ATGTAAAGTC  650 D6_19F9  1          11         21         31         41        50          |          |          |          |          |        |  ---------- ---------- ----GTAGCA ACAATCCCTG CCCTTTACTC   26 MLKL      AAGACAATTC CCTGGCAATT GTGTGTAGCA ACAATCCCTG CCCTTTACTC  350 F9_10     AAGACAATTC CCTGGCAATT GTGTGTAGCA ACAATCCCTG CCCTTTACTC  350 F9_11     CTCTACCCAA GATCTTGCTT GTTTCTTTCT AAGTTGCCTC TCTATCTAGC   76 MLKL      CTCTACCCAA GATCTTGCTT GTTTCTTTCT AAGTTGCCTC TCTATCTAGC  400 F9_10     CTCTACCCAA GATCTTGCTT GTTTCTTTCT AAGTTGCCTC TCTATCTAGC  400 F9_11     TTGCAGGATT TGAGTTGAGG AAAACACAGA CTTCCATG-- ----------  114 MLKL      TTGCAGGATT TGAGTTGAGG AAAACACAGA CTTCCATG-- ----------  438 F9_10     TTGCAGGATT TGAGTTGAGG AAAACACAGA CTTCCATGAG AGTTGAGTGT  450 F9_11     ---------- ---------- ---------- ---------- ----------  114 MLKL      ---------- ---------- ---------- ---------- ----------  438 F9_10     TGTTCCAGTT TGGAACAAGA GTCCACTATT AAAGAACGTG GACTCCAACG  500 F9_11     ---------- ---------- ---------- ---------- --AGTTTGGG  122 MLKL      ---------- ---------- ---------- ---------- --AGTTTGGG  446 F9_10     TCAAAGGGCG AAAAACCGTC TATCAGGGTG ATGGTTCACG TAAGTTTGGG  550 F9_11     AACTACGAGA GAAAAGACAG ACAGAGTCAA ATCTACAGCA TATCTCTCAC  172 MLKL      AACTACGAGA GAAAAGACAG ACAGAGTCAA ATCTACAGCA TATCTCTCAC  496 F9_10     AACTACGAGA GAAAAGACAG ACAGAGTCAA ATCTACAGCA TATCTCTCAC  600 F9_11 E8  1          11         21         31         41        50          |          |          |          |          |        |  CCTTTACTCC TCTACCCAAG ATCTTGCTTG TTTCTTTCTA AGTTGCCTCT    67 MLKL      CCTTTACTCC TCTACCCAAG ATCTTGCTTG TTTCTTTCTA AGTTGCCTCT   326 E8_32     ---------- ---------- ---------- ---------- AGTTGCCTCT   264 _R_E8_5   CTATCTAGCT TGCAGGATTT GAGTTGAGGA AAACACAGAC TTCCATGAGT   117 MLKL      CTATCTAGCT TGCAGGATTT GAGTTGAGGA AAACACAGAC TTCCATGAGT   376 E8_32     CT-------- --CAGGATTT CCATTGAGGA AAACACAAAC TTCCATGAGT   304 _R_E8_5        
